# Cellular localization of the FMRP in rat retina

**DOI:** 10.1042/BSR20200570

**Published:** 2020-06-15

**Authors:** Ping-Ping Zhang, Hui-Hui Yao, An-Hui Zha, Xing-Yue Liu, Ke-Yu Fan, Yue Xu, Hui-Yao Yuan, Lei Li, Lie-Cheng Wang

**Affiliations:** Department of Physiology, School of Basic Medical Sciences, Anhui Medical University, Hefei 230032, China

**Keywords:** amacrine cell, fragile X mental retardation protein, immunofluorescence double labeling, intrinsically photosensitive retinal ganglion cell, Müller cell

## Abstract

The fragile X mental retardation protein (FMRP) is a regulator of local translation through its mRNA targets in the neurons. Previous studies have demonstrated that FMRP may function in distinct ways during the development of different visual subcircuits. However, the localization of the FMRP in different types of retinal cells is unclear. In this work, the FMRP expression in rat retina was detected by Western blot and immunofluorescence double labeling. Results showed that the FMRP expression could be detected in rat retina and that the FMRP had a strong immunoreaction (IR) in the ganglion cell (GC) layer, inner nucleus layer (INL), and outer plexiform layer (OPL) of rat retina. In the outer retina, the bipolar cells (BCs) labeled by homeobox protein ChX10 (ChX10) and the horizontal cells (HCs) labeled by calbindin (CB) were FMRP-positive. In the inner retina, GABAergic amacrine cells (ACs) labeled by glutamate decarbonylase colocalized with the FMRP. The dopaminergic ACs (tyrosine hydroxylase marker) and cholinergic ACs (choline acetyltransferase (ChAT) marker) were co-labeled with the FMRP. In most GCs (labeled by Brn3a) and melanopsin-positive intrinsically photosensitive retinal GCs (ipRGCs) were also FMRP-positive. The FMRP expression was observed in the cellular retinal binding protein-positive Müller cells. These results suggest that the FMRP could be involved in the visual pathway transmission.

## Introduction

The fragile X syndrome (FXS) is the most common heritable form of intellectual disability caused by the decreased expression of the fragile X mental retardation protein (FMRP) [1]. The FMRP is an mRNA-binding protein mainly involved in intracellular RNA transport and translational regulation. The defective regulation of the local protein synthesis in the synapse results in altered synaptic plasticity and abnormal dendrite growth [2]. This condition suggests that the FMRP acts as a critical player in activity-dependent synaptic plasticity and thus in circuit formation and function [3,4]. Furthermore, loss of the FMRP leads to several deficits in visual subcircuits of the superior colliculus [5], and exhibit impairments in visual discrimination similar to those in *Fmr1^−/−^* mice and human FXS participants [6]. Although little is known about the FMRP and its possible role in vision, *Fragile X* premutation carriers have been found to have some visual perception impairments caused by the lack of the FMRP in the geniculo-striatal magnocellular visual pathway, which processes information about stimulus movement and cortical recipients [7]. Moreover, evidence shows that the FMRP regulates the translation of rhodopsin through post-translational modifications (phosphorylation in particular) [8]. Patients with FXS exhibit a wide range of vision integration dysfunctions that manifest in multiple modalities. These defects in visual sensory are a hallmark feature of many neurodevelopmental disorders associated with cerebral neuron immaturity [9,10], especially in the primary visual cortex [11]. Moreover, a report revealed that impairing the fragile X mental retardation 1 (*Fmr1*) gene expression by injecting morpholinos causes malformation of the zebrafish retina [12].

Several key proteins involved in synaptic transmission have been shown to be regulated by the FMRP. The defective regulation of local protein synthesis in the *Fmr1* knockout (KO) mice lowered the levels of GABAergic proteins, such as glutamic acid decarboxylase (GAD), and potassium channels [13–15]. The altered expression of the GABAR subunits redundancy was also linked to the FMRP loss-of-function in FXS [14,16,17]. One of the main pathways of the FMRP regulation is through the activation of the metabotropic glutamate receptor 5 (mGluR5) [5,18], which is expressed in the retina along with other mGluR [19–21]. Moreover, the FMRP is expressed in the retina, and the leading role of the FMRP is highlighted in the retinal function [22]. The absence of the FMRP correlates with the increase in the electroretinogram (ERG) b-wave, which mostly reflects ON-bipolar cell (BC) depolarization to light [23]. Nevertheless, the localization of the FMRP in different types of retinal cells has not been studied yet.

In the present study, by using double-labeled immunohistochemistry, we demonstrate that the FMRP is cell-type dependent in rat retina, including horizontal cells (HCs), several subtypes of amacrine cells (ACs), BCs, ganglion cells (GCs), and Müller cells.

## Experimental procedures

### Animals

A total of 20 male Sprague–Dawley rats (7–8 weeks old) were used in the present study. All were obtained from Anhui Medical University. In the supplementary data, two C57BL/6J male mice (7–8 weeks old, Anhui Medical University) and four *Fmr1* KO male mice (7–8 weeks old, The Jackson Laboratory, 003025) were used.

### Tissue preparation for immunocytochemistry

The retinas were prepared as previously described in detail [24]. In brief, the animals were deeply anesthetized with 20% urethane (10 ml/kg). The posterior eyecups were immediately fixed in fresh 4% paraformaldehyde in 0.1 M phosphate buffer (PB, pH 7.4) for 20 min and chilled sequentially in 10% (w/v), 20%, and 30% sucrose in 0.1 M PB at 4°C. The eyecups were then embedded in OCT (Sakura Finetek U.S.A., Inc., Torrance, Japan), frozen in liquid nitrogen, and sectioned vertically at 14-μm thickness on a freezing microtome (Leica, Nussloch, Germany). The sections were mounted on gelatin chromium-coated slides.

### DNA analysis and genotyping

Total DNA was isolated from the tail tissue that were collected from wild-type (WT) mice and *Fmr1* KO mice at approximately 2 weeks of age in the EP tube, cut and mark it. Add 80 μl NaOH (50 mmol/l), put in a metal bath at 99°C for 30 min, and add 40 μl Tris/HCl (1 mmol/l). After mixing, take 1 μl of each sample and add it to the reaction system (ddH_2_O + Buffer + dNTP + Taq enzyme + primer). *Fmr1* KO forward primer (5′-GTGGTTAGCTAAAGTGAGGATGAT-3′), and *Fmr1* KO reverse primer (5′-GTGGGCTCTATGGCTTCTGAGG-3′). WT forword primer (5′-ATCTAGTCATGCTATGGATATCAGC-3′), and WT reverse primer (5′-CTTGACTGTGCCGTTGAACT-3′). Polymerase chain reaction (PCR) was performed with the following protocol on a MyCycler Thermal Cycler™ (Bio-Rad, Hercules, CA, United States): 94°C for 5 min, 94°C for 30 s, 56°C for 30 s, 72°C for 45 s (35 cycles); 72°C for 10 min, and a final hold at 4°C. PCR products were run on 1% agarose gel. The *Fmr1* KO mouse amplicon was approximately 400 bp. The WT mouse amplicon was approximately 131 bp (Supplementary Figure 1). The amplicon was sequenced to determine the genotypes of the mouse. Select the required genotype *Fmr1* KO male mice and raise them separately.

### Immunocytochemistry

The procedures of immunocytochemistry were modified from Xu et al. [25]. Briefly, the retinal sections were preincubated in 0.1 M phosphate-buffered saline (PBS, pH 7.4), containing 6% normal donkey serum, 1% bovine serum albumin, and 0.2% Triton X-100 (PBST) for 2 h at 4°C. The rabbit polyclonal antibody against rat/mouse FMRP (corresponding to amino acid residues 549–569) (1:500 dilution, Abcam, ab17722, U.K.) was used for labeling the FMRP. The experiments were conducted by double labeling. All antibodies were mixed with PBST. The sections were combined with primary and secondary antibodies sequentially. The primary antibody has a unique locus for the secondary antibody that can emit immunofluorescence. Mouse anti-calbindin D-28k (CB) monoclonal antibody (1:2000 dilution, Swant, Bellinzona, Switzerland) and sheep anti-homeobox protein ChX10 (ChX10) polyclonal antibody (1:800 dilution, Abcam) were used for labeling HCs and BCs, respectively. The antibodies used for labeling the different subtypes of ACs were as follows: mouse anti-glutamate decarboxylase (GAD 65) monoclonal antibody (1:1000 dilution, Abcam) for GABAergic ACs, mouse anti-tyrosine hydroxylase (TH) monoclonal antibody (1:10000 dilution, Sigma, St. Louis, MO, U.S.A.) for dopaminergic ACs, and sheep anti-choline acetyltransferase (ChAT) polyclonal antibody (1:1000 dilution, Millipore, Billerica, MA, U.S.A.) for cholinergic ACs. Mouse anti-Brn3a monoclonal antibody (1:500 dilution, Santa Cruz Biotechnology, Santa Cruz, U.S.A.) and goat anti-homeobox protein melanopsin polyclonal antibody (1:500 dilution, Santa Cruz, U.S.A.) were used for labeling GCs and self-sensitized GCs, respectively. Mouse anti-cellular retinaldehyde-binding protein (CRALBP) monoclonal antibody (1:1000 dilution, Abcam) was used for labeling the Müller cells. The secondary antibodies used in this work were as follows: Alexa Fluor 488-conjugated donkey anti-rabbit IgG for labeling the FMRP; Alexa Fluor 555-conjugated donkey anti-mouse IgG for CB, GAD 65, TH, Brn-3a, and CRALBP; Alexa Fluor 555-conjugated donkey anti-goat IgG for melanopsin; and Fluor 555-conjugated donkey anti-sheep IgG for ChX10 and ChAT. The control experiments included the omission of the primary and/or secondary antibodies.

### Confocal laser scanning microscopy

The sections were scanned with a Leica SP2 confocal laser scanning microscope (ZEISS, LSM880+Airyscan, Germany) using a 40× immersion objective lens. For each double-labeling experiment, 30–36 sections on six different glass slides derived from three or four eyeballs were examined. Single optical sections were taken through the preparation and recorded digitally. To avoid any possible reconstruction stacking artifact, double labeling was precisely evaluated by sequential scanning on single-layer optical sections. The images were resized and adjusted for brightness and contrast in Adobe Photoshop to reproduce the original histological data.

### Western blot analysis

Western blot analysis was performed as described previously in detail [24]. Rat retinal extract samples (20 µl per lane) were loaded, subjected to 10% SDS/PAGE, and then transferred on to PVDF membranes. Non-specific binding was blocked for 2 h at room temperature in blocking buffer consisting of 20 mM Tris/HCl, pH 7.4, 137 mM NaCl, 0.1% Tween-20, and 5% non-fat milk. The blots were incubated with the anti-FMRP antibody (1:1000 dilution, Abcam) overnight at 4°C, followed by horseradish peroxidase-conjugated donkey anti-rabbit IgG (1:2000 dilution, Santa Cruz Biotechnology) for 1.5 h at room temperature, and finally visualized with the enhanced chemiluminescence automatic gel imaging analysis system (Peiqing Science and Technology, Shanghai, China). To estimate the molecular weight (MW) of the FMRP, a pre-stained protein mouse homeobox action (Tiangen, Beijing, China) was used.

## Results

**Figure 1:**
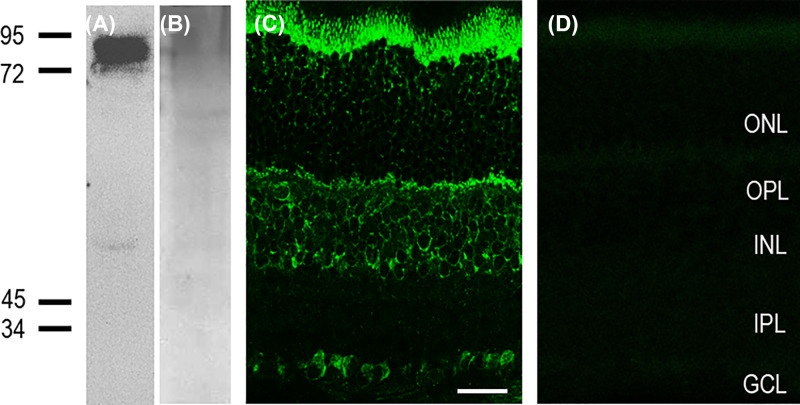
Expression of FMRP in rat retina (**A**) Western blot analysis of retinal homogenates for the FMRP antibody revealed a band at ∼80 kDa. (**B**) No band was detected when the FMRP antibody was absence in the incubated solution. (**C**) Confocal fluorescence microphotograph of a vertical section of the rat retina, labeled by FMRP. FMRP immunostaining is diffusely distributed through the whole retina. (**D**) Micrographs of a retinal vertical section, showing that no signal was detectable when the primary antibody for FMRP was omitted. Abbreviations: GCL, GC layer; INL, inner nuclear layer; IPL, inner plexiform layer; ONL, outer nuclear layer. Scale bar = 20 μm.

The specificity of the FMRP was examined using Western blot analysis. As shown in Figure 1A, in the rat retinal homogenates, the antibody against the FMRP recognized one band at ∼80 KDa, consistent with previous reports [26]. No band was found when the FMRP antibody was absent from the incubated solution (Figure 1B). These results suggest that the FMRP is indeed present in rat retina.

To determine the spatial expression pattern of the FMRP within the retina, the rabbit polyclonal antibody against rat FMRP was used. Figure 1C shows the general expression profile of the FMRP in the vertical section of adult rat retina. The immunoreaction (IR) of the FMRP was mainly observed in the inner retina, and the labeling was diffusely distributed throughout the full thickness of the retina, including the inner nucleus layer (INL), GC layer (GCL), and outer plexiform layer (OPL) but not the inner plexiform layer (IPL). Figure 1D shows that no signal was detectable when the primary antibody for FMRP was omitted. Compared with the general IR of the FMRP in Figure 1C, great differences were found in the expression of the antibody between them, and no FMRP-positive IR was found. Another negtive control, *Fmr1* KO mouse was used, and the same thing happened in the *Fmr1* KO mouse, no significant FMRP-positive IR was found in this mouse (Supplementary Figure 2, is the negtive control for FMRP antibody). These findings prove that the labeling signals in Figure 1C are FMRP-specific.

### Colocalization of the FMRP in the INL

**Figure 2:**
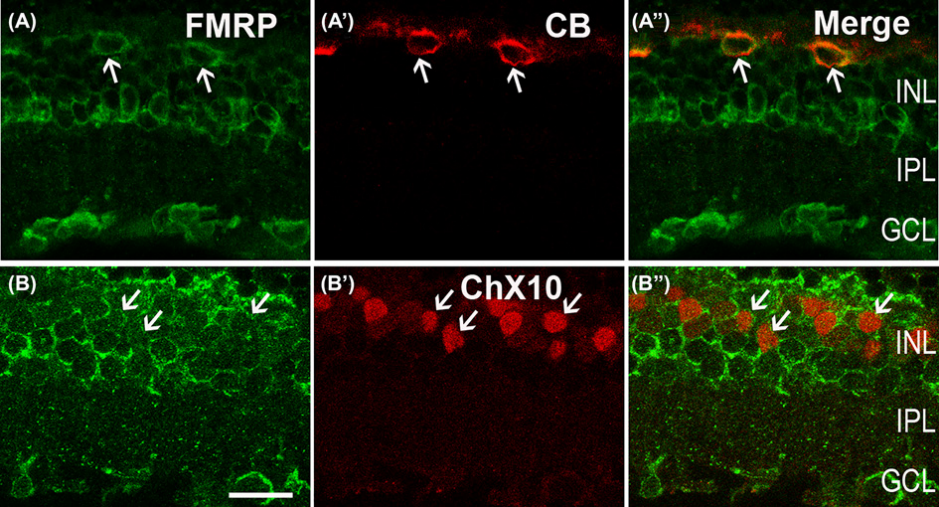
Confocal microphotographs, showing the distribution of FMRP in the outer retina(Notice! It should be replaced by Figure 1.) (**A,A**′′) Micrographs of a retinal vertical section, double-labeled by CB (**A′**) and FMRP (A,A′′). CB-labeled HCs (A′). The CB-positive somata (arrows) are co-labeled by FMRP (A,A′′). (**B,B′′**) Micrographs of a retinal vertical section, double-labeled by ChX10 (**B**′) and FMRP (B′′). ChX10 is a marker for BCs. Note that labeling for FMRP is detected in most of ChX10-positive BCs (arrows). Scale bars = 20 μm.

To determine the FMRP expression in the outer retina, or specifically, the fraction near the OPL, which actually is the synaptic connection layer, the colocalization of the FMRP with the HC and BC markers was performed. Figure 2A,A′′ show the vertical sections of the rat retina double-immunolabeled with the antibodies against the FMRP and CB, a marker for rodent HCs [27,28]. As revealed in Figure 2A′′, the merged image of Figure 2A,A′ in the INL labeling for the FMRP and CB, the CB-positive HCs were clearly stained by the FMRP, especially in the membrane. The BCs labeled with ChX10, a specific pan-BC marker [29] were uniformly ChX10-positive in the outermost portion of the INL but not in the axons (Figure 2B′). When the sections were co-stained with the FMRP (Figure 2B′′), the soma of the BCs was prominently embedded within the membrane. Thus, the BCs were also expressed FMRP especially in the cytoplasm, and possibly associated with the plasma membrane.

In the inner part of the INL, as shown in the vertical section of a rat retina (Figure 1C), the FMRP has a clear expression in this region. According to their positions and shapes, these FMRP-positive cells could be ACs, displaced ACs, or GCs. ACs are composed of numerous subtypes [30]. Special markers for the different subtypes of ACs were used to determine the identity of FMRP-labeled ACs.

The GABAergic ACs are observed to constitute the majority of ACs in the mammalian retina, and they include dopaminergic and cholinergic ones stained by TH and ChAT, respectively. Thus, the double-labeling experiments were performed to explore the identities of the neurons immunolabeled by the FMRP in the inner retina. Intriguingly, GABAergic drugs (e.g., baclofen) were examined for neurodevelopmental disorders, which prominently include the FXS and related autism spectrum disorders (ASDs) [31].

**Figure 3:**
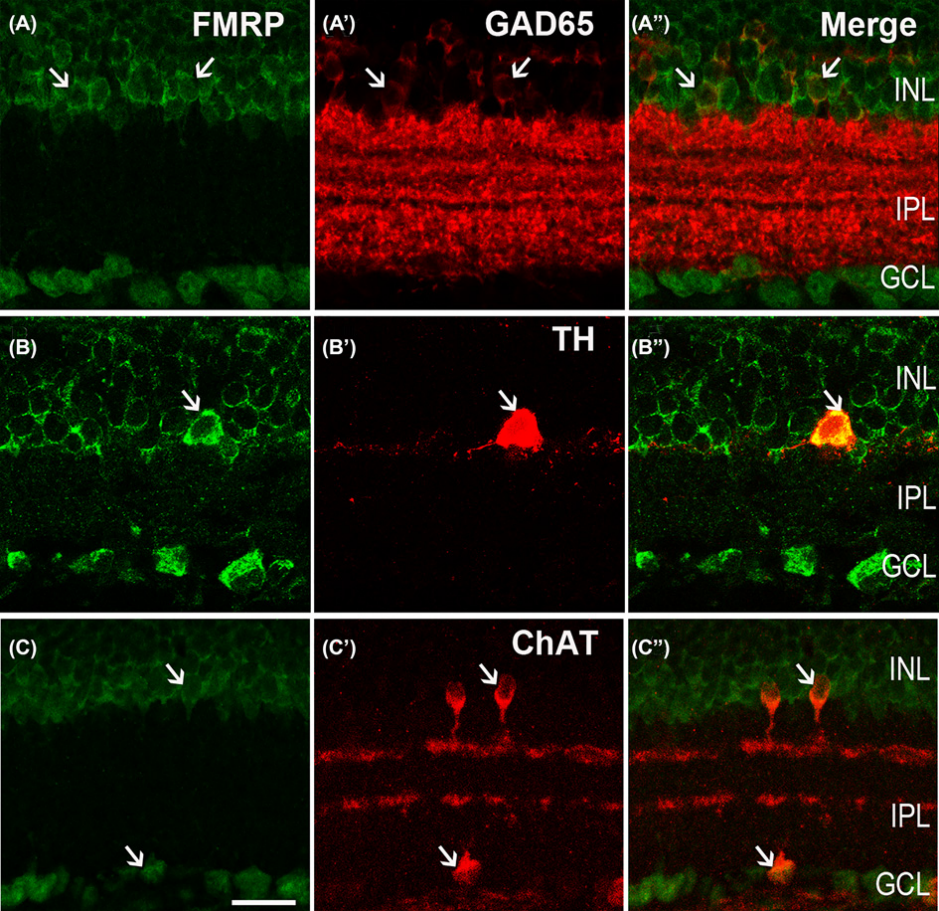
Confocal microphotographs, showing the distribution of FMRP in different AC subtypes Double-labeled elements (red for GAD 65, TH and ChAT; green for FMRP) appear yellowish, and arrows indicate some of double-labeled cells. (**A,A**′′) Micrographs of a retinal vertical section, double-labeled by GAD 65 and FMRP. (A′′) is the merged image of (A) (labeling for FMRP) and (**A**′) (labeling for GAD 65). Note that the GABAergic ACs in the innermost part of the INL are GAD 65-labeled, and also stronly labeled numerous neuronal processes of these cells in the IPL (A′). Almost all GAD 65-postive GABAergic ACs are labeled by FMRP (A′′). (**B,B**′′) Micrographs of a retinal vertical section, double-labeled by TH and FMRP. The soma and processes of the dopaminergic AC is strong labeled by TH (**B′**,B′′), and the soma is clearly labeled by FMRP (B′′). (**C**–**C**′′) Micrographs of a retinal vertical section, showing the co-localization of ChAT and FMRP. ChAT-labeled mirror-symmetric cholinergic ACs and their processes, forming two bands in the IPL. We can find that the somata of cholinergic ACs in INL and GCL were both FMRP-positive (C). Scale bar = 20 μm.

The GABARs are expressed in the retina, which is largely a neural tissue, and the GABAergic ACs constitute approxiamtely half of all those in the mammalian retina [30,32] labeled by GAD 65 [24]. Therefore, the anti-GAD 65 antibodies were used to label the GABAergic ACs. Figure 3A,A′′ show the micrographs of a vertical section of the rat retina double labeled by the antibodies against GAD 65 and FMRP, respectively. The peripheral cytoplasm of the scattered cells in the INL (arrows) labeled by GAD 65 were GABAergic ACs. The numerous neuronal processes of these cells in the IPL were also labeled by GAD 65 (Figure 3A′). As shown in the merged image (Figure 3A′′) of Figure 3A (labeling for FMRP) and 3A′, most of the GABAergic cells in the INL were co-labeled by FMRP. Therefore, the presence of the colocalization of FMRP and GAD 65 confirmed that FMRP is expressed in GABAergic ACs.

The GABAergic ACs constitute the majority of ACs in the mammalian retina, include dopaminergic and cholinergic ones. Figure 3B′ shows a TH-labeled dopaminergic AC (arrow) in an adult rat retinal section, which is located in the inner part of the INL and emits its process mainly into the outmost border of the IPL. As shown in the merged image (Figure 3B′′) of Figure 3B,B′, the somata of the TH-positive cell were strongly colocalized (arrow) with FMRP. As previously reported [33], the somata of the ChAT-labeled cholinergic ACs were situated either in the INL (arrow) or in the GCL (arrow, Figure 3C′) and their processes forming two distinct narrow fluorescence bands in the IPL. Thus, somata of these cholinergic ACs, including in the GCL, were FMRP positive (Figure 3C,C′′), but their processed bands were FMRP-negative.

### Colocalization of FMRP in the GCL

**Figure 4:**
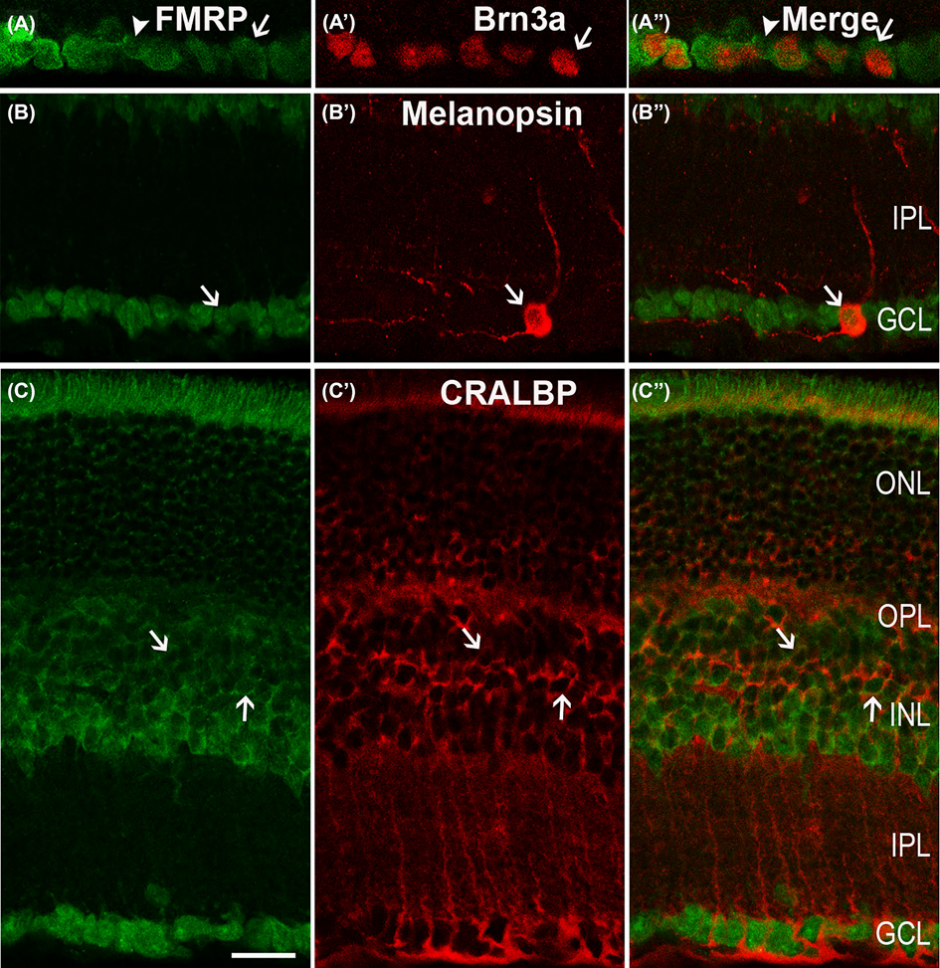
Confocal microphotographs, showing the distribution of FMRP in GCL and Müller cells Duble-labeled elements (red for Brn3a, melanopsin and CRALBP; green for FMRP) appear yellowish, and arrows indicate some of double-labeled cells. (**A,A**′′) Micrographs of a retinal vertical section, double-labeled by Brn3a and FMRP. GCs are clearly labeled by Brn3a (**A**′). FMRP immunoreactivity is observed in all Brn3a (arrows). Meanwhile, some FMRP-positive cells in the GCL are not immunoreactive for Brn3a (arrowheads in A,A′′), which could be displaced ACs or Brn3a-negative GCs. (**B**-**B**′′) Micrographs of a retinal vertical section, double-labeled by melanopsin and FMRP, showing that melanopsin-positive somata (arrows) are co-labeled by FMRP, which demonstrates FMRP is expressed in ipRGCs, especially in the soma of ipRGCs. (**C-C**′′) Micrographs of a retinal vertical section, double-labeled by CRALBP, a Müller cell maker, and FMRP. Note that FMRP imunostaining is observed in somata of CRALBP-positive Müller cells. Scale bar = 20 μm. Abbreviation: ipRGC, intrinsically photosensitive retinal GC.

As previously mentioned, the cells in the GCL that were labeled by FMRP could be the GCs or displaced ACs. To clarify these cells, a double-labeling experiment was further conducted with FMRP and Brn3a, a specific GC marker [34]. The results (Figure 4A,A′′) revealed that the Brn3a-positive cells in the GCs expressed FMRP because the two label colors (somata, stained red by Brn3a; membranes, stained green by FMRP) perfectly fit into each other. However, some Brn3a-positive cells were not labeled (arrowheads in Figure 4A,A′′), and these cells could be the displaced ACs or Brn3a-negative GCs.

The intrinsically photosensitive retinal GCs (ipRGCs) are a special GC subtype that accounts for a small number of GCs involved in setting the suprachiasmatic nucleus circadian clock [35,36] and they are Brn3a-negative GCs. Melanopsin polyclonal antibodies were used for labeling the ipRGCs. The anti-melanopsin experiment (Figure 4C′) demonstrated that melanopsin-positive GCs only accounted for a small population of the GCs and that the fluorescence appeared in the membrane and the cytoplasm of the ipRGCs. The double-labeling experiment (Figure 4B,B′′) with FMRP and melanopsin showed that the FMRP-stained cells found within the GCLs were also positive for melanopsin. That is, FMRP was also expressed in the ipRGCs, especially in the soma of the ipRGCs.

### Colocalization of FMRP in the Müller cells

From the panoramic distribution of the FMRP, we observed regularly arrayed fiber-like processes and trunks spanning the entire neural retina, with their characteristic end-feet in the GCL, which corresponds with the distribution of the Müller cells. Therefore, CRALBP was used to mark the rat Müller cells, including the processes and somata [37]. As shown in Figure 4C,C′′, the overlay of the corresponding paired images of the double labeling of the FMRP and CRALBP appeared yellowish in the vertical sections of rat retina. The FMRP was also expressed in the soma of the Müller cells (arrows), but the main trunks, the distinguishable parallel processes, and the end-feet in the GCL could not be labeled with the FMRP.

## Discussion

The FMRP is an mRNA-binding regulator encoded by the *Fmr1* gene. The FMRP was detected in the retina in previous experiments, but its localizations and functions have not yet been studied [22]. In this work, we demonstrated that the FMRP was expressed diffusely in the inner and outer rat retina, especially in the INL and GCL, through immunohistochemistry doubled-labeling experiments. According to its location, we speculated that it could be involved in the transmission and integration of visual signals to some extent.

**Figure 5:**
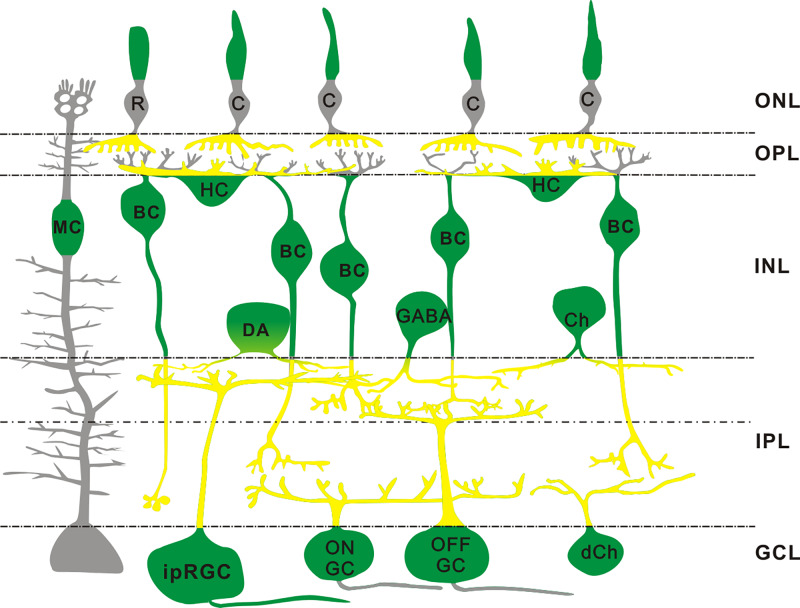
Schematic diagram summarizing the expression of FMRP in the rat retina FMRP is clearly expressed in somata of HCs and BCs, definitely in the process of BCs. In addition, somata of GABAergic ACs, including dopaminergic and cholinergic ACs, somata of GCs, MCs and ipRGCs are FMRP-positive (all in green). The dendrites of BCs in the OPL are not labeled by FMRP (all in gray). Because of diffuse labeling in the OPL and IPL, terminals and dendrites of photoreceptors, processes of ACs and dendrites of HCs and GCs may be FMRP-positive (all in yellow). Abbreviations: C, cone; Ch, cholinergic AC; DA, dopaminergic AC; dCh, displaced cholinergic AC; GABA, GABAergic AC; MC, Müller cell; R, rod.

According to the previous study [22], FMRP is detected in photoreceptors inner/outer segments, but not clear in the soma. Based on our results, the FMRP was expressed in HCs (marked by CB) and BCs (marked by ChX10) in the INL (Figure 2). GABAergic neurons (marked by GAD 65) were also labeled by the FMRP (Figure 3A′′). The FMRP IRs were observed in almost all dopaminergic neurons (marked by TH) and cholinergic ACs (marked by ChAT) (Figure 3B′′,C′′) as well as in GCs (marked by Brn3a) and ipRGCs (marked by melanopsin) in the GCL (Figure 4A′′,B′′). The Müller cells, which were stained by CRALBP, were also co-labeled with the FMRP (Figure 4C′′). The schematic diagram in Figure 5 summarizes the colocalization of the FMRP in rat retina.

BCs are intermediate neurons in the signaling pathway of the mammalian vision system. They receive signal inputs from the photocatalyst (cones and rods) and transmit them to the non-dendritic cells and GCs. In conclude, BCs play a key role in the processing of visual information and the longitudinal transmission of information, after the integration of the information. Retinal HCs have an important role in the processing of visual information and the transverse transmission of information. We speculate that the FMRP may be involved in the retinal information processing in different ways, probably by modulating the radial flow of visual signals and the lateral interaction mediated by BCs and HCs, as visual sensory impairments have been described in mental deficiency (MD) and ASD, including FXS [38,39]. This protein has been proved to be ubiquitously produced in mammalian tissues with high levels in the brain [40] and localized predominantly in cytosolic light and heavy membranes. Furthermore, the retina is part of the central nervous system with a common embryonic origin, the mechanisms in the signal transmission from outer to inner retina were described in the ERGs of the *Fmr1^−/−^* mouse [23,41], and were shown to have very early onset in development [41].

In fact, MD and ASD have been linked to brain dysfunction caused by axonal development and maturation of dendritic spines [9,10,42] that lead to synaptic defects [11,43,44]. At present, impaired perception is attributed to changes in the brain's phenotype. According to researches, in patients with FXS, visual integration is particularly affected, with changes in temporal processing capabilities, reduced high-frequency visual stimulation contrast sensitivity, and sensitivity to dynamic and static images [38,39]. These visual sensory defects are related to the immature development of neurons in the brain [9], especially in the main visual cortex [9]. So far, no data on the retinal perception light signal has been collected in patients with MD, ASD, and FXS. Although the retinal conduction pathway is localized in the cerebral cortex, the retina is part of the central nervous system and shares a neurodevelopmental origin with the mesencephalon [45,46]. Therefore, retinal and brain nerve cells show great similarities in neurotransmitters, highly differentiated neuron configurations and functional procedures [47]. Based on these similarities, we speculate that the perception function of the retina itself may change accordingly.

## Highlights

1. The labeling of FMRP was diffusely distributed throughout the full thickness of retina, including GCL, INL as well as OPL, but not IPL.
2. In the outer part of the INL, FMRP are clearly expressed in BCs and HCs.
3. In the inner part of the INL, FMRP are positive in the soma of GABAergic, dopaminergic, and cholinergic ACs.
4. In the GCL, FMRP are expressed in the GCs including ipRGCs.
5. The expression of FMRP is seen in the Müller cells.

## Supplementary Material

Supplementary Figures S1-S2

## Abbreviations

AC: amacrine cell
ASD: autism spectrum disorder
BC: bipolar cell
CB: calbindin
ChAT: choline acetyltransferase
ChX10: homeobox protein Chx10
CRALBP: cellular retinaldehyde-binding protein
ERG: electroretinogram
Fmr1: fragile X mental retardation 1
FMRP: fragile X mental retardation protein
FXS: fragile X syndrome
GAD 65: glutamic acid decarboxylase
GC: ganglion cell
GCL: GC layer
HC: horizontal cell
INL: inner nucleus layer
IPL: inner plexiform layer
ipRGC: intrinsically photosensitive retinal GC
IR: immunoreaction
KO: knockout
MD: mental deficiency
OPL: outer plexiform layer
PB: phosphate buffer
PCR: polymerase chain reaction
WT: wild-type
