# Supplementary material for: Cellular localization of the FMRP in rat retina
Source: Biosci Rep. 2020 Jun 15;40(6):BSR20200570. doi: 10.1042/BSR20200570 (PMC7295639; doi:10.1042/BSR20200570)
Supplement: Supplementary Figures S1-S2 [file BSR-2020-0570_supp.pdf]

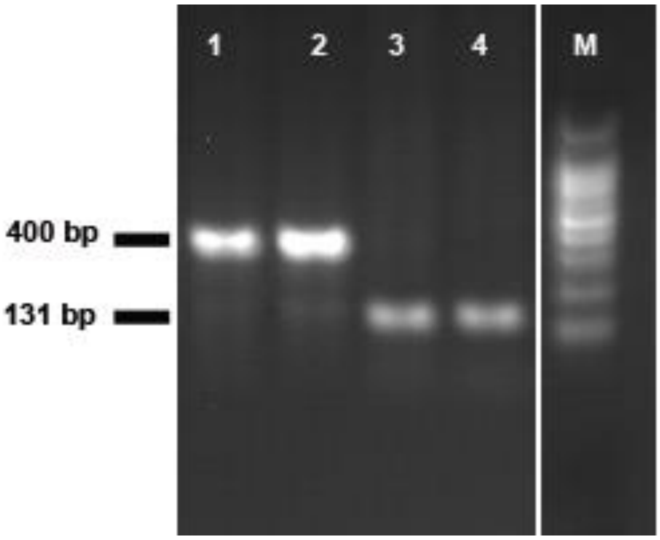
1
2
3
4
M
400 bp
131 bp

Supplementary Figure 1. Genotype analyses of *Fmr1* KO mice. Ethidium bromide-stained agarose gel with PCR reactions from four different mice tails (1,2,3 and 4). Mice 1 and 2 are *Fmr1* KO mice with a DNA double-strand length of 400bp. Mice 3 and 4 are WT mice with a DNA double-strand length of 131bp. M: marker.

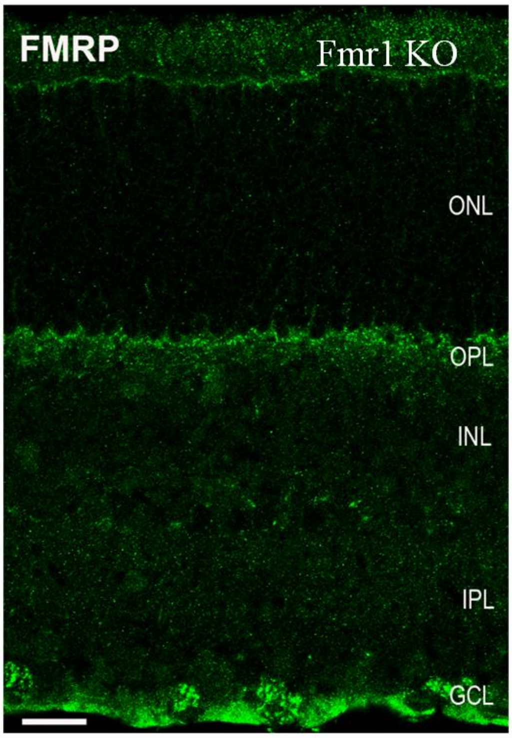
FMRP
Fmr1 KO
ONL
OPL
INL
IPL
GCL

Supplementary Figure 2. Expression of FMRP in the retina of *Fmr1* KO mouse. Confocal fluorescence microphotograph of a vertical section of the *Fmr1* KO mouse retina, labeled by FMRP. FMRP immunostaining isn't exist through the whole retina. Scale bar = 20 μm.
